# Supplementary material for: Hoverfly (Eristalis tenax) pursuit of artificial targets
Source: J Exp Biol. 2023 Feb 27;226(4):jeb244895. doi: 10.1242/jeb.244895 (PMC10088529; doi:10.1242/jeb.244895)
Supplement: Supplementary information [file jexbio-226-244895-s1.pdf]

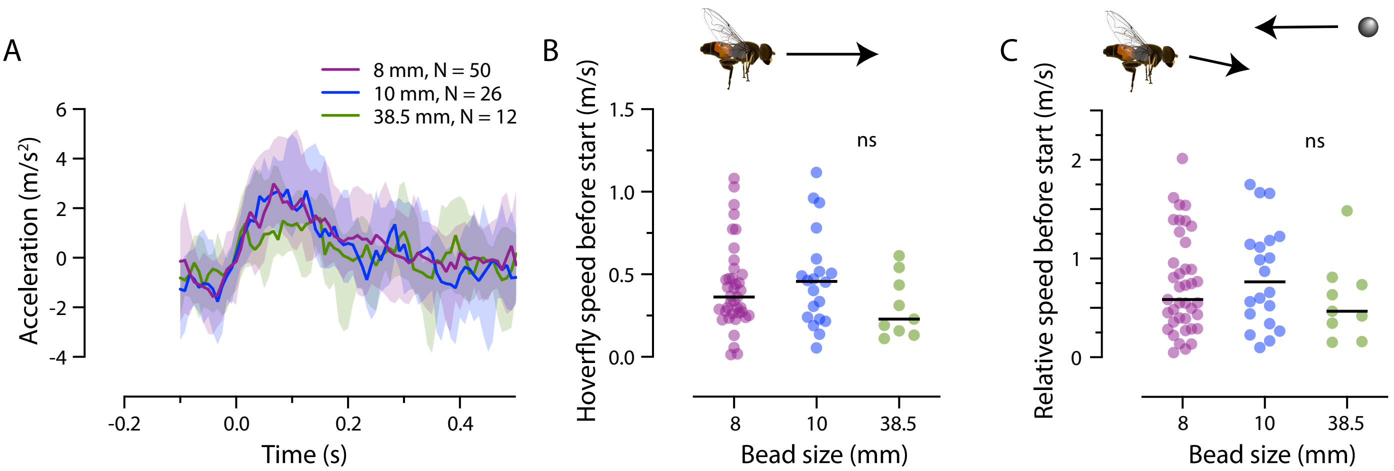

Fig. S1. ***Hoverflies accelerate rapidly after pursuit start***

A) The hoverflies' acceleration at pursuit start. We smoothed the flight speed using a span of 10% of the total number of data points before calculating the acceleration. The data show the median +/- interquartile range, color coded according to bead size. There was no significant effect of bead size (using a mixed-effect model). B) The hoverfly flight speed 100 ms before pursuit start. C) The relative speed between the hoverfly and the bead 100 ms before pursuit start. The data in panels B and C were analyzed using Kruskal-Wallis tests.

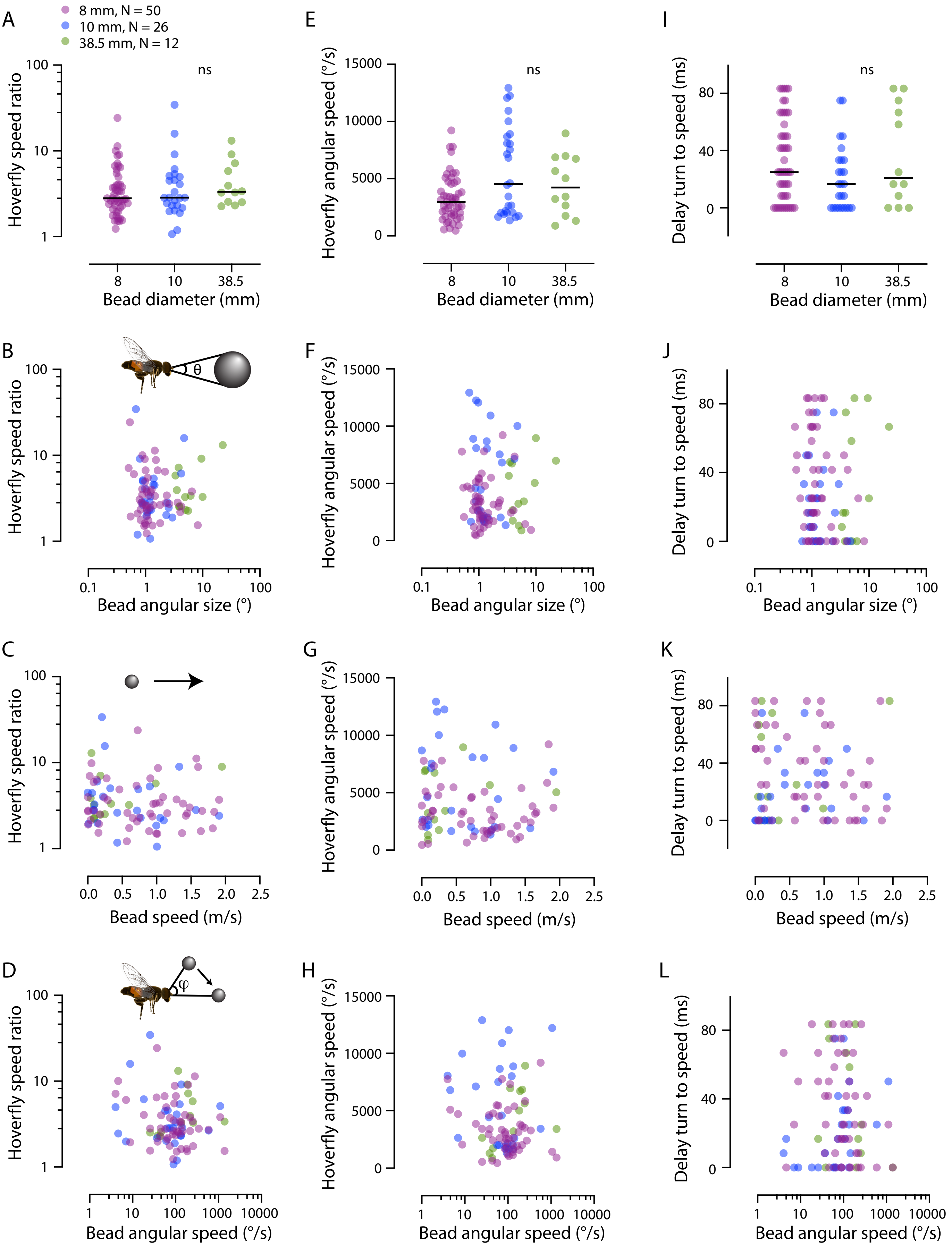

Journal of Experimental Biology • Supplementary information

Fig. S2. Hoverfly behavior at pursuit start does not depend on the bead

Figure S2 aims to investigate how the hoverfly might adjust its flight behavior at pursuit start based on the bead. For this purpose, we display the hoverfly speed ratio (left column), the hoverfly angular speed (middle column), and the delay between the turn and speed increase (right column), as a function of the bead's physical size (top row), the bead's angular size (2nd row), the bead's translational speed (3rd row), and the bead's angular speed (bottom row). As evident from the data, there is no clear correlation between the hoverfly's behavior at pursuit start and any input from the bead. As explained in the main text, this is contrary to published accounts of pursuit behavior in the field (e.g. Collett and Land, 1978). The panels show the following: A) The hoverfly speed ratio following pursuit start, as a function of the bead diameter. B) The hoverfly speed ratio following pursuit start, as a function of the size of the bead's angular image. C) The hoverfly speed ratio, as a function of the bead's translational speed. D) The hoverfly speed ratio, as a function of the bead's angular speed. E) The hoverfly angular speed at pursuit start, as a function of bead diameter. F) The hoverfly angular speed at pursuit start, as a function of the bead's angular size. G) The hoverfly angular speed as a function of bead's speed at pursuit start. H) The hoverfly angular speed at pursuit start, as a function of the bead's angular speed. I) The delay between the hoverfly peak angular speed and peak speed ratio, as a function of bead diameter. J) The delay as a function of the bead's angular size. K) The delay as a function of the bead's speed. L) The delay as a function of the bead's angular speed. In all panels, the data are color coded according to bead size. The data in panels A, E, I were analyzed using Kruskal-Wallis tests.

Journal of Experimental Biology • Supplementary information

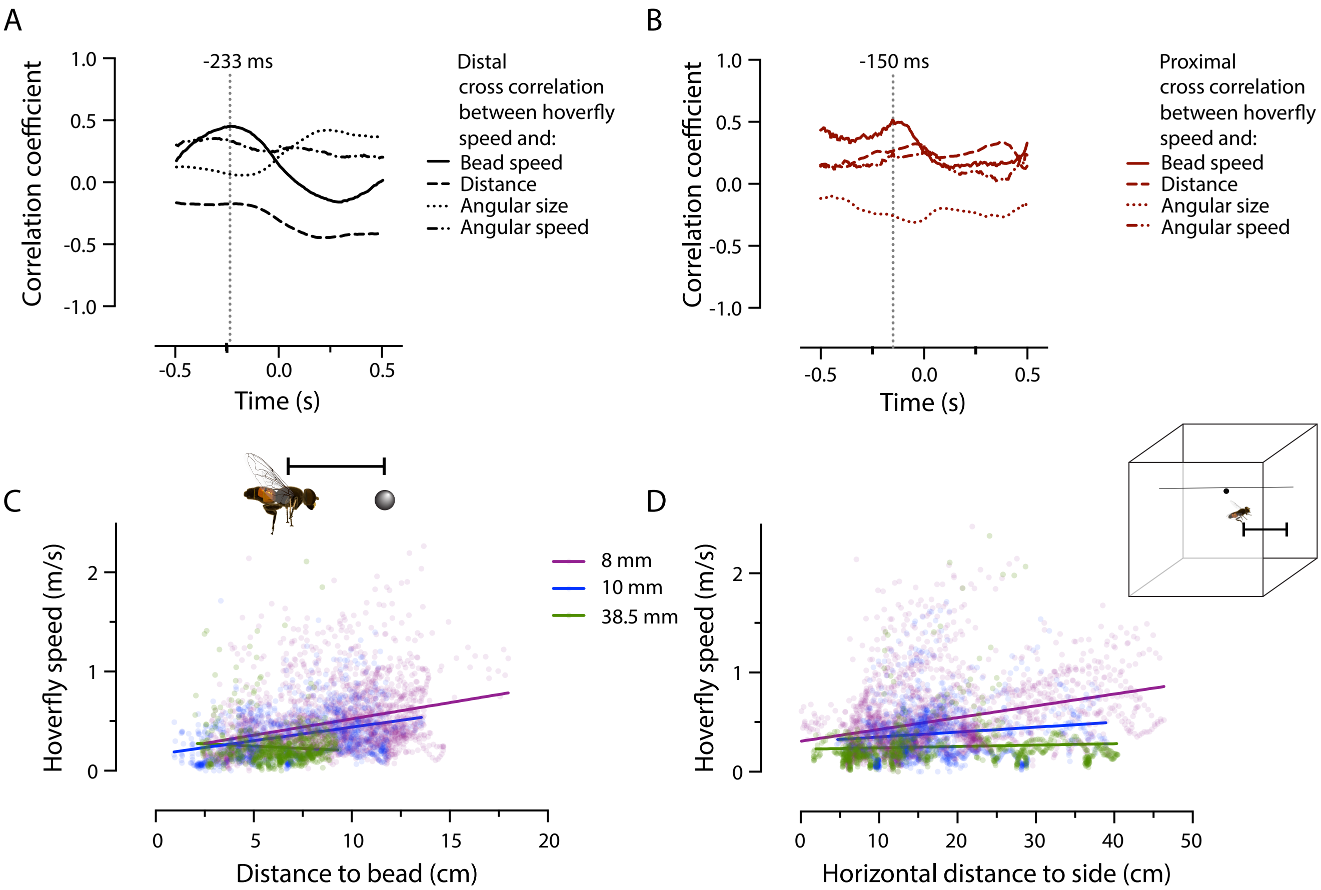

Fig. S3. The hoverfly flight speed is best correlated with the bead speed

A) The cross correlation between the hoverflies' translational speed and the beads' translational speed (solid line), the distance to the bead (dashed), the bead's angular size (dotted) and angular speed (dash-dotted) during the first 1 s of the distal stage of the pursuit. Peak correlation was found with the bead speed at -233 ms (dotted vertical line). B) Cross correlation between the hoverflies' translational speed and the beads' speed (solid line), the distance (dashed), the angular size (dotted), and the angular speed (dash-dotted), during the first 1 s of the proximal stage of the pursuit. Peak correlation was found with bead speed at 150 ms (dotted vertical line). C) The hoverflies' instantaneous flight speed as a func-tion of the distance to the bead speed 58 ms previously (based on peak correlation, dashed line, panel B), during the first 1 s of the proximal stage. The lines show linear regression (R^2 = 0.096, 0.11 and 0.006 for the three bead sizes). D) The hoverflies' instantaneous flight speed as a function of the distance to either the left or the right wall of the arena, whichever was closest, during the first 1 s of the proximal stage. The lines show linear regression (R^2 = 0.10, 0.016 and 0.004 for the three bead sizes).

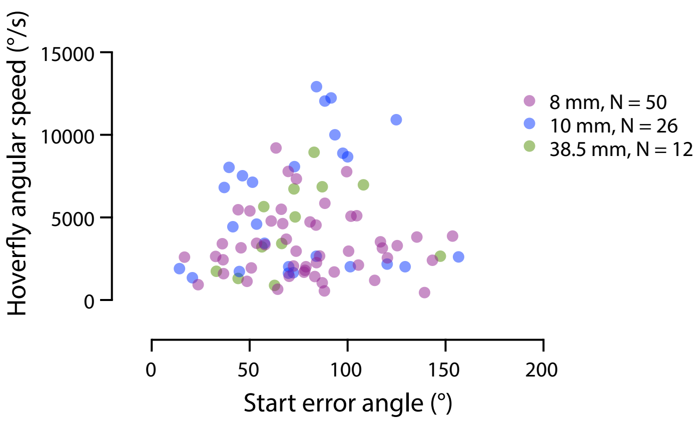

Fig. S4. Hoverfly angular speed at pursuit start does not depend on the error angle to the bead

The hoverfly angular speed, as a function of the error angle to the bead, both at pursuit start. The data are color coded according to bead size.

Journal of Experimental Biology • Supplementary information

Table S1. Hoverfly cohorts and their age when placed in the flight arena

Cohort #	Age of hoverflies at start
1	0-1 days
2	5 months
3	2-3 months
4	3 weeks
5	4 days

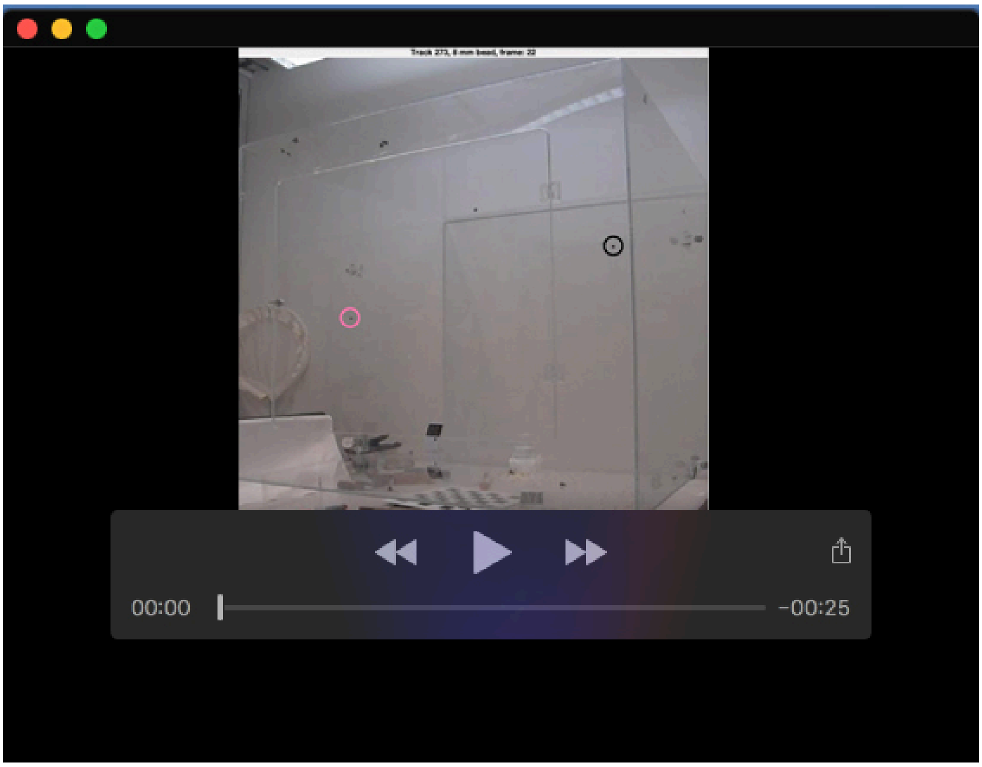

Movie 1. Pursuit of a 6 mm diameter bead

The movie shows an example of a male *E. tenax* (pink circle) pursuing a 6 mm diameter bead (black circle). Pursuit start is highlighted with a yellow star.

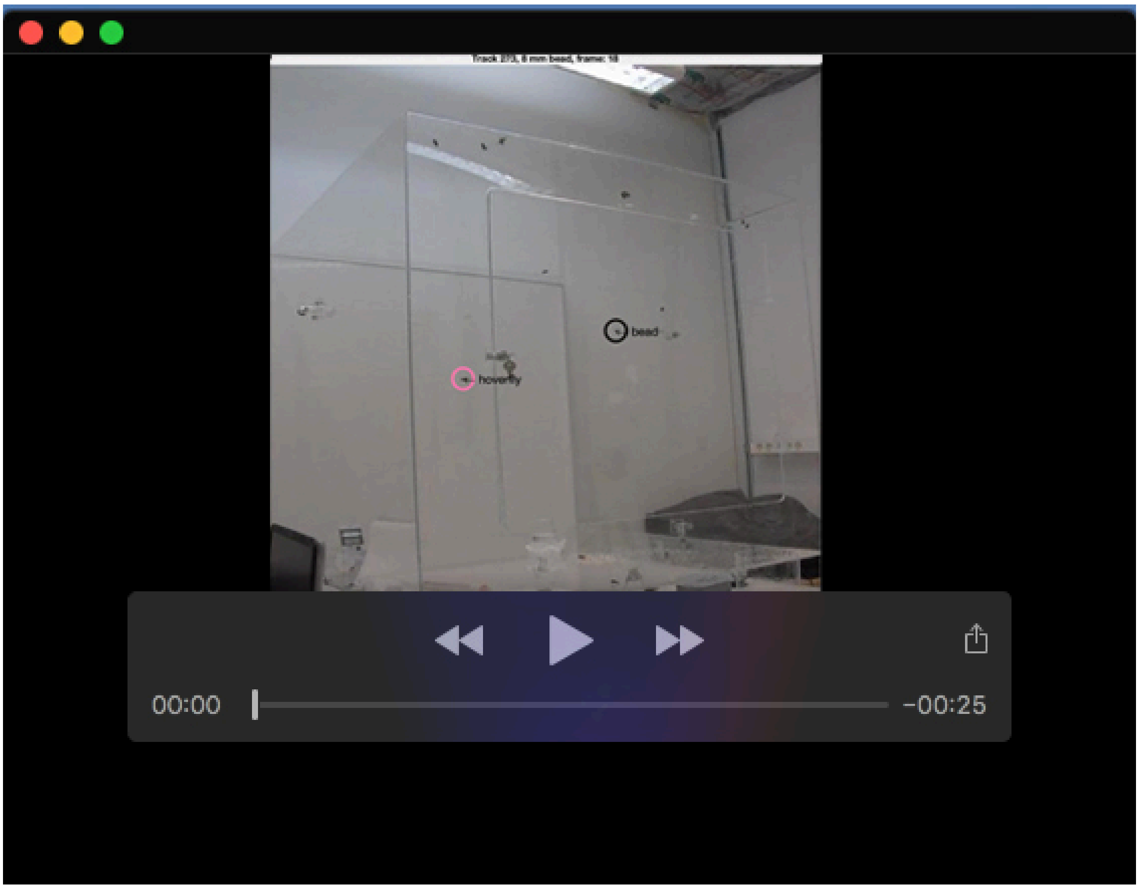

Movie 2. Pursuit of an 8 mm diameter bead, camera 1 view

The movie shows an example of a male *E. tenax* (pink circle, which becomes purple during the proximal stage) pursuing an 8 mm diameter bead (black circle) programmed to move at 1 m/s. Pursuit start is highlighted with a yellow star. This pursuit is also shown in Figure 2B.

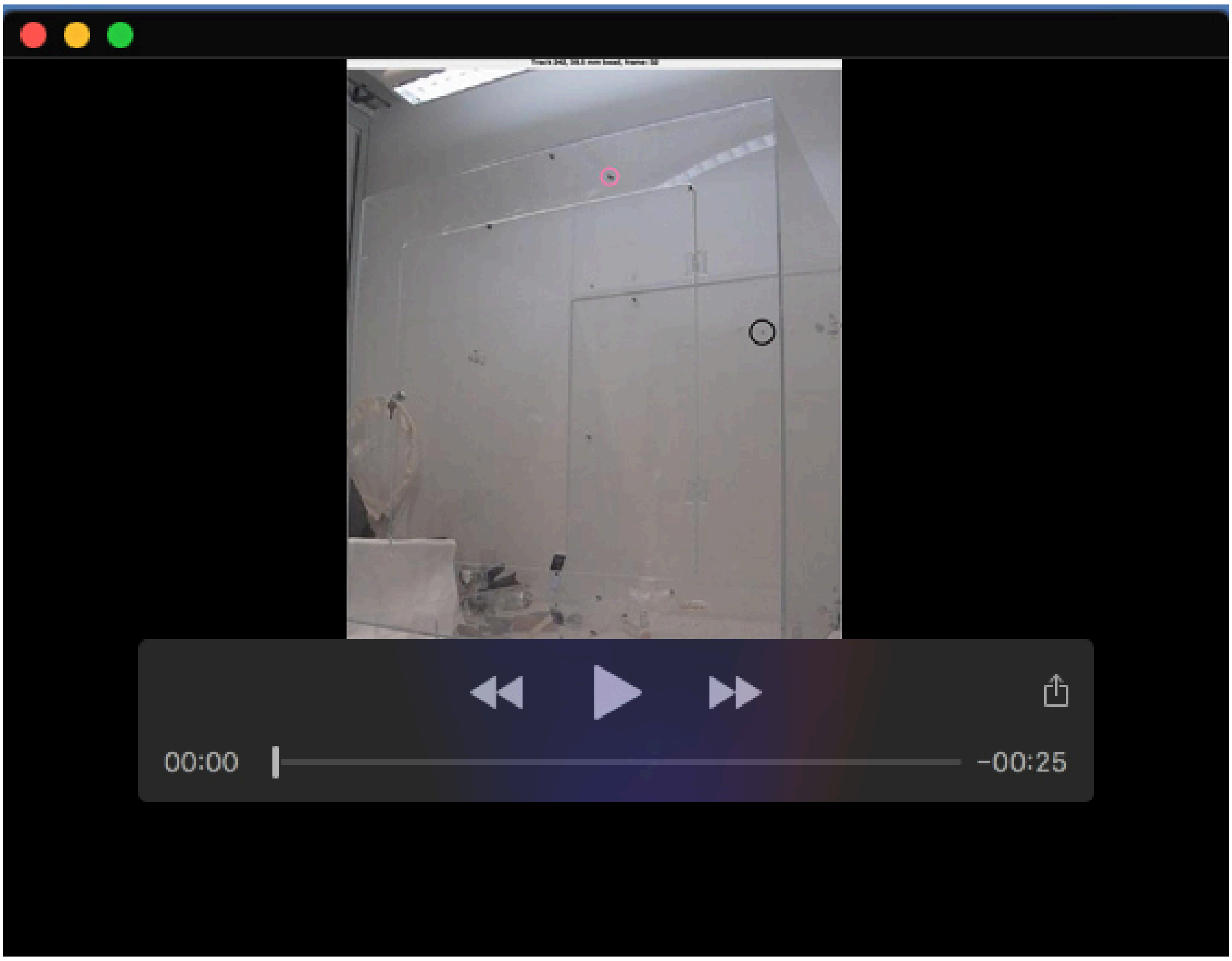

Movie 3. Pursuit of an 8 mm diameter bead, camera 2 view

The movie shows the same male *E. tenax* (pink circle, which becomes purple during the proximal stage) pursuing an 8 mm diameter bead (black circle) programmed to move at 1 m/s, seen from the second camera. Pursuit start is highlighted with a yellow star. This pursuit is also shown in Figure 2B.

Reference cited in supplementary material

Collett, T. S. and Land, M. F. (1978). How hoverflies compute interception courses. *J Comp Physiol A* **125**, 191-204.
